# The Crosstalk of PTGS2 and EGF Signaling Pathways in Colorectal Cancer

**DOI:** 10.3390/cancers3043894

**Published:** 2011-10-14

**Authors:** Dingzhi Wang, Dianren Xia, Raymond N. DuBois

**Affiliations:** 1 Departments of Cancer Biology, The University of Texas MD Anderson Cancer Center, Houston, TX 77030, USA; E-Mails: dwang11@mdanderson.org (D.W.); dxia@mdanderson.org (D.X.); 2 Gastrointestinal Medical Oncology, The University of Texas MD Anderson Cancer Center, 1515 Holcombe Blvd., Houston, TX 77030, USA

**Keywords:** PTGS2, COX-2, EGFR, colorectal cancer

## Abstract

Colorectal cancer (CRC) is now the second-leading cause of cancer deaths in the USA. Colorectal cancer progression and metastasis depends on the orchestration of the aberrant signaling pathways that control tumor cell proliferation, survival and migration/invasion. Epidemiological, clinical, and animal studies have demonstrated that prostaglandin-endoperoxide synthase 2 (PTGS2) and epithelial growth factor (EGF) signaling pathways play key roles in promoting colorectal cancer growth and metastasis. In this review, we highlight major advances in our understanding of the roles of PTGS2 and EGF signaling in colorectal cancer.

## 1. Introduction

Colorectal cancer (CRC) is the fourth most common malignant neoplasm and the second leading cause of cancer deaths in the USA [1]. Depending on the primary colorectal carcinoma's stage, liver metastases occur in 20% to 70% of patients and lung metastases in 10% to 20% of patients. Unfortunately, distant metastases are the major cause of death for patients with advanced CRC because standard treatments for metastatic CRC are not as effective as needed, resulting in extremely low 5-year survival rates. Thus, a better approach for this disease must include prevention and personalized targeted therapy. Clearly, understanding the molecular mechanisms responsible for CRC progression and metastases will lead to novel strategies for the development of new agents for CRC prevention and treatment.

CRC is a heterogeneous disease. At least three major forms of CRC have been described: hereditary, sporadic, and colitis-associated CRC. A large body of evidence indicates that genetic mutations, epigenetic changes, chronic inflammation, diet and lifestyle (smoking, physical inactivity, and alcohol) are risk factors for CRC. Patients with familial adenomatous polyposis (FAP), due to a germ-line mutation in one allele of the tumor suppressor gene adenomatous polyposis coli (*APC*), have a near 100% risk of developing CRC by the age of 40 if untreated. Hereditary nonpolyposis colorectal cancer (HNPCC), which is due to inherited mutations in genes for DNA mismatch repair such as *MLH1*, *MSH2*, and *MSH6*, is responsible for approximately 2 to 7 percent of all diagnosed cases of CRC. Moreover, patients with inflammatory bowel disease (IBD) face an increased lifetime risk for developing CRC. Colitis-associated CRC affects individuals at a younger age than the general population. Epidemiologic and animal studies provide evidence that a high fat diet can be associated with an increased risk for cancers, including CRC [2]. Metabolism of arachidonic acid, a major ingredient in animal fats, by prostaglandin-endoperoxide synthase enzymes provides one mechanism for the contribution of dietary fats and chronic inflammation to carcinogenesis.

The development of CRC involves the accumulation of genetic alterations and progressive changes in signaling pathways that control tumor initiation, dysregulation of mitosis and apoptosis of tumor cells, angiogenesis, and metastatic spread. The alterations in multiple pathways such as WNT, prostaglandin-endoperoxide synthase 2 (PTGS2, also called as COX-2), EGF receptor (EGFR) and RAS are known to play a major role in CRC progression. Canonical WNT/CTNNB1 signaling is a potent initiator of human colorectal carcinogenesis and is modulated by several key molecules, including the catenin beta 1 (CTNNB1, also called as β-catenin) oncogene, the adenomatous polyposis coli (APC) tumor suppressor, AXIN, and glycogen synthase kinase-3 beta (GSK-3B). Somatic loss of *APC* occurs in about 85% of sporadic colorectal adenomas and carcinomas [3-5]. Our group was the first to report that COX-2 expression is elevated in human CRC [6]. Multiple follow-up studies revealed that PTGS2 (COX-2) levels are elevated in other premalignant and malignant solid tumors, including stomach, esophagus, liver, pancreas, head and neck, lung, breast, and prostate cancer [7]. Epidemiological, clinical, and animal studies provide evidence demonstrating that PTGS2 (COX-2) plays a key role in promoting CRC progression, whereas inhibition of PTGS2 (COX-2) prevents tumor growth and improves overall survival [8]. Moreover, clinical and animal studies have also revealed an important role of the EGFR signaling in a subgroup of CRC [9-12].

Animal models are essential tools in mechanistic studies of the pathogenesis of disease and drug development in CRC research. Although none of the existing mouse models mimic human CRC precisely, the chemically induced models such as carcinogen azoxymethane (AOM) and genetically altered models such as the *Apc^Min/+^* mouse have been most commonly used. For example, *Apc^Min/+^* mouse carried a point mutation at the *Apc* gene is considered to be a model for human FAP and is also used as a pre-malignant model for human sporadic CRC since the *APC* mutation occurs very early with a high frequency (85%) in human sporadic CRC. In this review, we focus on recent insights into the roles of the PTGS2 (COX-2) and EGFR signaling pathways in coordinately promoting colorectal carcinogenesis.

## 2. PTGS2 (COX-2) Signaling in Colorectal Cancer

**Figure 1.**
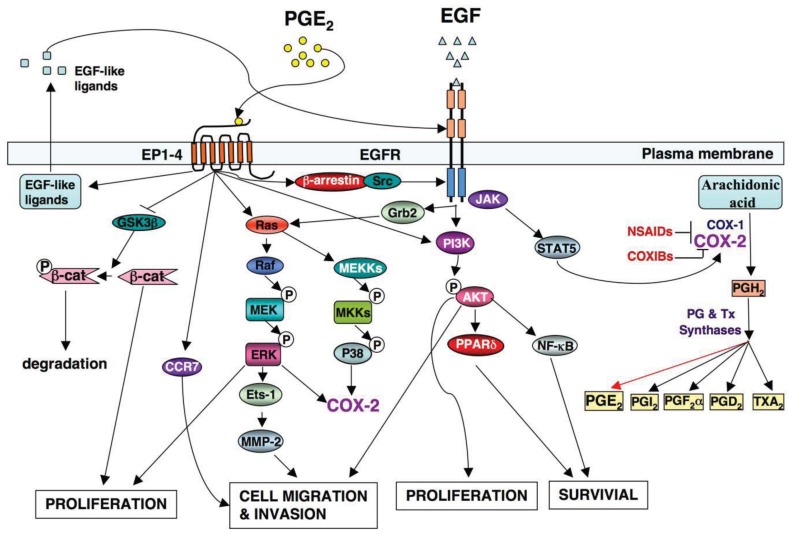
The crosstalk between COX-2 and EGF signaling pathway in CRC.

A significant effort has been made in the development of novel drugs for both cancer prevention and treatment over the past decades. One group of compounds found to decrease the risk of CRC includes nonsteroidal anti-inflammatory drugs (NSAIDs), which primarily target the PTGS1 and PTGS2 (COX-1 and COX-2) (Figure 1).

An increasingly large body of evidence from population-based studies and clinical trials has shown that regular use of NSAIDs over a 10–15 year period reduces the relative risk of developing CRC by 40–50% [13]. In particular, regular aspirin use is much more effective at reducing risk in the group of patients whose colon tumors expressed high levels of PTGS2 (COX-2) [14] and improves overall survival after the diagnosis of CRC at stage I, II and III, again in individuals with tumors that overexpress PTGS2 (COX-2) [15]. Although selective PTGS2 (COX-2) inhibitors (COXIBs) and non-selective NSAIDs are still among the most promising chemo-preventive agents for this disease, long-term use of high doses of COXIBs and non-selective NSAIDs (except for aspirin) is currently not recommended because of the unacceptable cardiovascular side effects in certain patients, especially in those individuals with a history of atherosclerotic heart disease [16]. However, a recent report that retrieved all existing epidemiologic studies (case control and cohort studies) from 1980 showed that the meta-analysis of independent estimates from 72 studies provided no evidence that daily use of the celecoxib increased the relative risk of cardiovascular disease [17]. Moreover, another cohort study examined cardiovascular outcomes in approximately 1.4 million patients receiving NSAIDs or COXIBs showed that there was, again, no risk observed with most compounds, including celecoxib [18]. Therefore, it is necessary to further assess the relative risks and benefits of NSAIDs or COXIBs in different clinical settings such as people with or without vascular disease and patients with adenomas.

It has been hypothesized that some of the adverse cardiovascular effects related to COXIBs and NSAID use are associated with a global reduction in prostanoid production [19]. There are at least two ways to avoid these undesired side effects. One way to use low doses of COXIBs and NSAIDs with other anti-tumor agents may provide more effective therapeutic and chemopreventive effects with decreased cardiovascular effects known to occur with COXIBs. Another is to only target PTGS (COX)-derived prostanoids that mediate the tumor-promoting effects of PTGS2 (COX-2).

### 2.1. PTGS2 (COX-2) Regulation

PTGS2 (COX-2) is an immediate-early response gene normally absent from most cells but is induced mainly at sites of inflammation in response to inflammatory stimuli including pro-inflammatory cytokines such as IL1A/B, IFNG, and TNF (TNFalpha) produced by inflammatory cells as well as tumor promoters such as tetradecanoyl phorbol acetate (TPA) and RAS *in vitro* and *in vivo* [20,21]. By contrast, PTGS1 (COX-1) is constitutively expressed in a broad range of cells and tissues and its expression remains constant under most physiological or pathological conditions. COX-1 generally contributes to maintenance of the gastric mucosa, regulation of renal blood flow in the afferent vessels of the kidney, and regulation of platelet aggregation.

PTGS2 (COX-2) expression is regulated in both transcriptional and post-transcriptional levels. It is well established that the PTGS2 (COX-2) transcription can be regulated by various transcription factors such as NFKB1 (NF-κB), C/EBP, CREB, NFAT, AP-1, and PPAR. PTGS2 (COX-2) mRNA is also modulated by post-transcriptional mechanism via AU-rich regions in its 3′-untranslated region (3′UTR). The RNA-binding proteins Hu antigen R (HuR) and tristetraprolin (TTP) bind AU-rich elements in the 3′UTR of PTGS2 (COX-2) to stabilize or to decay its mRNA *in vitro,* respectively [22]. Recently, microRNAs (miRNAs) have been suggested to silence PTGS2 (COX-2) expression by translational repression and/or degradation of its mRNA via 3′UTR *in vitro* [23,24]. In addition to the inflammatory microenvironment, a hypoxic environment also induces PTGS2 (COX-2) expression in colorectal tumor cells via HIF-1α factor *in vitro* [25].

### 2.2. Mechanism(s) of PTGS2 (COX-2) in CRC

The first evidence linking PTGS2 (COX-2) to carcinogenesis emerged from studies on CRC [6]. Several subsequent reports confirmed that PTGS2 (COX-2) expression is elevated in approximately 50% of human colorectal adenomas and 85% of adenocarcinomas [6,26,27] and is associated with a worse survival among CRC patients [28]. Direct molecular evidence that PTGS2 (COX-2) plays a key role in colorectal carcinogenesis was obtained from studies in animal models. Genetic studies demonstrate that deletion of PTGS2 (COX-2) gene results in decreased tumor formation in both small intestine and colon of *Apc^Min^* mice [29] as well as in *ApcΔ^716^* mice [30]. In contrast, overexpression of PTGS2 (COX-2) in mouse intestinal epithelium promotes tumor growth in AOM-treated mice [31].

The PTGS2 (COX-2) enzyme converts arachidonic acid into an unstable PGH_2_ intermediate that is further metabolized to five structurally related prostanoids, including prostaglandins (PGs) such as PGE_2_, PGD_2_, PGF_2〈_α, PGI_2_ and thromboxane A_2_ (TxA_2_) via specific PG and TX synthases (Figure 1). Since PTGS2 (COX-2) is induced mainly at sites of inflammation in response to inflammatory stimuli, inflammatory prostaglandins are produced in many cells including colonic epithelial cells. Among prostanoids, pro-inflammatory PGE_2_ plays a predominant role in promoting tumor growth. PGE_2_ is the most abundant PG found in various types of human malignancies including colon, lung, breast, head and neck cancer and is often associated with a poor prognosis [32-35]. A urinary PGE_2_ metabolite (PGE-M) has been used as a promising biomarker for CRC [36,37] and other cancer patients [38,39]. The levels of PGE_2_ in tumor tissues depend on the relative rates of PTGS2 (COX-2)/PGE synthase-dependent biosynthesis and hydroxyprostaglandin dehydrogenase 15-(NAD) (HPGD, also called as 15-PGDH)-dependent degradation. HPGD (15-PGDH) is highly expressed in normal tissues but is ubiquitously lost in human colon, gastric, lung, and breast cancer [40-43]. Loss of HPGD (15-PGDH) in these tumor tissues results in increased endogenous PGE_2_ levels. Multiple lines of evidence from mouse models of CRC demonstrate that PGE_2_ mediates the effects of PTGS2 (COX-2) on promoting colorectal tumor growth. PGE_2_ treatment reverses NSAID-induced regression of small intestinal adenomas in *Apc^Min/+^* mice [44] and elevated endogenous PGE_2_ levels via loss of Hpgd (15-Pgdh) inhibit the anti-tumor effects of celecoxib in the AOM mouse model [45]. Direct evidence that PGE_2_ promotes tumor growth comes from our and other studies showing that PGE_2_ treatment dramatically increased both small and large intestinal adenoma burden in *Apc^Min/+^* mice and significantly enhanced AOM-induced colon tumor incidence and multiplicity [46,47]. Furthermore, elevated endogenous PGE_2_ via genetic deletion of *Hpgd (15-Pgdh)* promotes colon tumor growth in *Apc^Min/+^* and AOM mouse models [48]. In contrast, inhibition of endogenous PGE_2_ via genetic deletion of PGE_2_ synthase (*Ptges*) suppresses intestinal tumor formation and growth in *Apc^Min/+^* and AOM models [49]. The central role of PGE_2_ in colorectal tumorigenesis has been further confirmed by evaluating mice with a homozygous deletion of individual PGE_2_ receptors [50-52]. Given that PGE_2_ appears to play a dominant role in carcinogenesis, it is conceivable that identification of the downstream targets of PGE_2_ may elucidate the mechanisms by which PTGS2 (COX-2) promotes CRC progression and metastasis.

## 3. EGF Signaling in Colorectal Cancer

EGF is a member of the EGF family, comprising EGF, transforming growth factor αlpha (TGFA), amphiregulin, heparin-binding EGF (HB-EGF), cripto, betacellulin, epigen, epiregulin, and neuregulin. The gastrointestinal tract expresses at least six members, including EGF, TGFA, amphiregulin, heparin-binding EGF, and neuregulin. The members of EGF family exert their biological effects in autocrine or/and paracrine manner by binding to their cognate cell surface receptors, which belong to the type I receptor tyrosine kinase (TK) family, including EGFR, ERBB2 (HER2), ERBB3 (HER3), and ERBB4 (HER4). These receptors share similar structure with an extracellular binding domain, a transmembrane domain, and an intracytoplasmic domain with TK activity and regulator functions. All have an intrinsic TK domain except for ERBB3 (HER3). After binding with EGF and EGF-like ligands, they form homodimers or heterodimers, which results in activation of the receptor TK by autophosphorylation. The activation of the receptors recruits different adaptor molecules to trigger multiple downstream signal transduction pathways such as RAS-RAF-MAPK, PI3K-AKT, JAK-STAT, and phospholipase C cascades. The signal cascades activated by different EGF ligands drive various transcription factors to nucleus and regulate different cellular responses such as proliferation, survival, migration, differentiation, and apoptosis [53]. For example, the PI3K-Akt cascade mediates EGFR signaling in stimulating proliferation of normal intestinal epithelial cells and CRC cells *in vitro* [54,55].

Aberrant expression and/or activities of EGF family members and their receptors have been reported in a number of solid tumor malignancies including CRC [56-59]. For example, the expression and activities of EGFR as well as its ligands TGFA and HB-EGF are positive correlated with formation of aberrant crypt foci (ACF) in the colon of patients [60], indicating their role in very early stage of colorectal carcinogenesis. Moreover, the expression of EGFR and its ligands is often elevated in tumors as compared to matched normal tissues [61] and appears to be associated with malignant progression, with stronger expression in carcinoma than in adenoma [62]. Since the expression of EGFR directly correlates with the ability of human CRC cells to the liver metastasis [63], EGFR may be used as potential metastatic biomarker for CRC patients [10,64]. Thus, EGF signaling contributes to colorectal carcinogenesis at various stages.

In animal studies, EGFR activity is elevated in the adenomas and has been associated with intestinal adenoma growth in *Apc^Min/+^* mice [65]. Disruption of EGFR signaling by either genetic mutation or its kinase inhibition blocks the formation and growth of ACF, microadenomas, and adenomas as well as the growth of established tumors in *Apc^Min/+^* and AOM-treated mice [66-68]. Specific overexpression of EGFR in mouse mammary gland induces epithelial cell transformation [69]. These results suggest that EGFR signaling is required for CRC formation and progression.

## 4. The Crosstalk between PTGS2 (COX-2) and EGF Signaling Pathways in CRC

Although specific antibodies to EGFR signaling pathway have been developed and approved by FDA for treatment of patients with metastatic CRC, there has been limited success using these antibodies as a single agent or adjuvant treatment with irinotecan or cytotoxic agents. This is likely due to redundant pathways or the activation of other compensatory ones that negate the therapeutic efficacy of these drugs. For example, the presence of a *KRAS* mutation and the mutant p110 subunit of PI3K in the patients with advanced CRC results in a clinical resistance to anti-EGFR therapy such as monoclonal antibodies cetuximab and panitumumab [70,71] because both KRAS and PI3K are downstream targets of the EGFR pathway (Figure 1). Moreover, our group has demonstrated that PGE_2_ induces colorectal carcinoma cell migration and invasion through an EGFR-PI3K-AKT signaling *in vitro* [72]. Since PGE_2_ transactivates EGFR via an intracellular signaling pathway [73] and PGE_2_ can also directly activate the PI3K-AKT pathway in an EGFR-independent manner (Figure 1), we hypothesize that activation of PGE_2_ signaling could affect clinical response to the anti-EGFR therapy. Thus, additional research is needed to address the questions of how multiple signaling pathways coordinately regulate CRC metastasis and how combining treatments for targeting multiple pathways are selected.

Both PTGS2 (COX-2) and EGFR pathways are activated in many human cancers [74]. Understanding how two pathways orchestrate tumor progression will provide a significant advance in the cancer field. Multiple lines of evidence demonstrate that a cross talk of PTGS2 (COX-2) and EGFR pathways synergistically promotes CRC progression and metastasis (Figure 1). The observations that EGFR activity is increased in a PTGS2 (COX-2) transgenic mouse and forced expression of PTGS2 (COX-2) in human CRC cells stimulates cellular proliferation through induction of EGFR [31,75] indicate that EGFR is a downstream target of PTGS2 (COX-2). Our laboratory has demonstrated that PGE_2_ induces CRC cell migration and invasion through an EGFR-PI3K-Akt signaling *in vitro* [72]. Subsequently, we found that PGE_2_ induction of an EP4/ARRB1/SRC complex was critical in transactivating the EGFR to induce downstream AKT signaling and stimulated CRC cell migration *in vitro* as well as metastatic spread of disease to the liver *in vivo* [73]. The SRC-EGFR pathway also mediates PGE_2_-induced human hepatocellular carcinoma cell invasion *in vitro* [76]. These studies reveal that PGE_2_ transactivates the EGFR via an intracellular mechanism. Other groups reported that PGE_2_ transactivation of EGFR depended on the extracellular release of EGF-like ligands such as amphiregulin and TGFA in CRC cell lines [77-79]. PGE_2_ may also regulate EGF signaling via canonical WNT pathway based on the evidence that PGE_2_ activates WNT signaling via TCF4 transcription factors by stabilizing CTNNB1 (β-catenin) in CRC cells [80] and Apc deficiency is associated with increased Egfr activity in the intestinal enterocytes *in vivo* [65]. Conversely, it has been recognized that EGF signaling upregulates PTGS2 (COX-2) expression. For instance, EGFR activation results in PTGS2 (COX-2) expression at both mRNA and protein levels in a rat intestinal epithelial cell and colon cancer cells *in vitro* [81,82]. Activation of EGFR induces PTGS2 (COX-2) expression through multiple pathways, including STAT5, SRC, and MAPK14 (P38 MAPK) [83,84]. In contrast, inhibition of EGFR signaling by its TK inhibitor (gefitinib) blocks the expression of PTGS2 (COX-2) induced by AOM in a mouse model [67,68]. These findings support the notion that such positive feedback loop may amplify the activity of both PTGS2 (COX-2) and EGFR pathways, which in turn results in coordinate promotion of CRC progression and metastasis.

In addition to the cross talk of PGE_2_ and EGFR pathways, PGE_2_ is able to induce proliferation of colon and lung cancer cells through activating MAPK in an EGFR-independent manner *in vitro* [85,86]. Moreover, PGE_2_ promotes colon tumor cell survival by upregulation of BCL2 or/and activating NFKB1 (NF-κB) *in vitro* [87,88]. PGE_2_ may also induces cell migration and invasion by upregulation of matrix metalloproteinase 2 (MMP2) via an ERK-ETS1 cascade in pancreatic cancer cell lines [89] and upregulation of CCR7 via PTGER2 (EP2) and PTGER4 (EP4) in breast cancer cell lines [90]. On the other hand, EGFR signaling also induces colon cancer cell proliferation, survival, and migration/invasion through multiple pathways in a PTGS2 (COX-2)-independent manner.

Researchers have been investigating whether inhibiting both the EGFR and PTGS2 (COX-2) signaling pathways at lower doses could yield additive effects on blocking tumor growth and the spread of metastatic disease. Preclinical studies supports the notion that combined treatment of NSAIDs and EGFR TK inhibitors is more effective than either single agent alone in several models. In colorectal carcinoma cells, blocking both PTGS2 (COX-2) and the ERBB2 (HER2) pathways synergistically reduced tumor growth [91]. In soft agar and xenograft assays, the combined treatment of a PTGS2 (COX-2) selective inhibitor, an EGFR tyrosine kinase inhibitor, and a protein kinase A antisense construct markedly reduced proliferation and angiogenesis of human colon and breast cancer cells [92]. Similarly, combined treatment with inhibitors of both pathways significantly prevents the formation of polyps by over 96% and completely inhibits polyp growth in *Apc^Min/+^* mice [93,94]. These findings suggest that the inhibition of both PTGS2 (COX-2) and EGFR pathways may provide a better therapeutic strategy. Hence, we feel that it will be essential to examine the use of PTGS2 (COX-2) selective inhibitors as potential agents in combination with EGFR tyrosine kinase inhibitors in clinical trials.

## 5. Conclusions

Both EGFR and PTGS2 (COX-2) represent promising targets for cancer prevention and treatment. Specific targeting of EGFR is already used in patients with metastatic colon and lung cancer. For example, the FDA has approved two monoclonal antibodies (cetuximab and panitumumab), which target EGFR, for treatment of patients with metastatic CRC and two EGFR tyrosine kinase inhibitors (gefitinib and erlotinib) for treatment of patients with metastatic non-small cell lung cancer. Given that both EGFR and PTGS2 (COX-2) pathways orchestrate to promote tumor initiation, progression, angiogenesis, and metastasis, it is conceivable that combined targeting to both PTGS2 (COX-2) and EGFR pathways will yield better clinical outcome in CRC prevention and treatment.
